# Dr. John Milton Duff

**Published:** 1904-06

**Authors:** 


### OBITUARY.

Dr. John Milton Duff, of Pittsburg, died at his home, May 14, 1904, of septicemia, aged 55 years. He was a soldier of the civil war, having enlisted in Company B, 107th Regiment, Pennsylvania volunteers. After the war he studied medicine and graduated from Jefferson Medical College in 1874. He practised his profession in Pittsburg and became professor of gynecology in the Western Pennsylvania Medical College. He was also obstetrician-in-chief and abdominal surgeon to the Rhineman Hospital, consulting surgeon and gynecologist to the South Side Hospital; Fellow of the American Academy of Medicine; Fellow of the American Association of Obstetricians and Gynecologists, and was chairman of the section on obstetrics and gynecology in the American Medical Association in 1893. Dr. Duff was a man of forceful character, amiable disposition and a physician of distinction.

